# Imagination Power

**Published:** 1874-09

**Authors:** 


### HALL'S

### JOURNAL OF HEALTH

AND

### MISCELLANY.

VOL. XXI.—SEPTEMBER, 1874.—NO. IX.

### IMAGINATION POWER.

MANY persons are sick merely from the effect of imagination or habit, the old woman, for example, who had been bed-ridden for years with her "rheumatics." She was left alone one summer day at the farm house with both front and rear doors open. She saw a mad bull tearing across the pasture in the direction of the house, and feeling that it was "neck or nothing" she bounced out of that bed in double quick time, barred both the doors, and never complained of rheumatism afterward.

Many cases are given in the medical journals of persons who have been bitten by dogs, and have afterwards taken to foaming at the mouth, shuddering at the sight of water, and imitating a dog's bark, imagining that these are signs of hydrophobia, and have actually died in convulsive agonies, the facts being that mad dogs don't foam at the mouth, never bark, and can drink water "like a fish." So far from running about in desperation as if a hundred thousand dilapidated tin pans were tied to their caudal prolongation, really mad dogs are the quietest individuals in the world, make no noise, seem to want to be alone, keep their mouths shut, the distinctive symptoms of such madness being great restlessness, pawing the face and eating their own fresh dejections. Mad dogs do not run after people, but will snap at anyone who comes in their way, especially individuals of their own kind, they seem to trot or run as if looking at something straight ahead. Infinite mischief is done in publishing cases of persons becoming hydrophobic one, ten, and even twenty years after the last remembered biting of a dog, the reporters of such tales not having taken pains to inquire if there has not been a later biting. Such monstrous absurdities are well calculated to keep nervous persons, who have been once bitten by a dog, in a state of wearying uneasiness all their lives long afterwards. If a person has been bitten by a dog supposed to be mad, the animal should be put in a quiet room and let alone, only slipping in food and drink from time time; if really mad death will take place in a day or two, and if not mad there will be a complete recovery.

Only one bite in twenty of dogs supposed to be mad is fatal.

A dog in New Jersey last year bit a woman, and ran under the sofa; her husband dragged him out by the tail, held him up and whipped him; in this position the dog got a chance of biting his master, who died in a few days of hydrophobia; this shows that the state of mind of the animal gives virulence to the bite. A mother nursing her infant child was thrown into a sudden, a tempestuous rage; as soon as it was over, she finished nursing the child, which was at once taken with convulsions; the state of mind of the mother imparting virulence to the milk of her bosom. A lady saw at a distance a window sash falling immediately on the ends of the fingers of her little grandchild; the child's fingers were crushed—those of the grandmother were similarly affected. This is given on the testimony of the distinguished Dr. Brown Sequard. The lesson is, seek to control the imagination and to guard against intense mental excitement by habituating the mind to take a calm and measured and deliberate view of all the circumstances of life. Intelligent people should bear these things in mind. The greatest throat swabber of the age began to think he had sore throat too, and swabbed himself every day; and when he died his throat was found to have been as well as anybody's.

The best way to escape imaginative diseases is to be just as busy as you can in doing something useful, profitable, or good; to have the mind fully employed in some commendable object; it is thus that the washerwoman is often happier than the wife of the millionaire; the hod carrier, than he who has "retired from business."

